# Intravitreal Injections of Cord Blood Platelet-Rich Plasma in Dry Age-Related Macular Degeneration: Regenerative Therapy

**DOI:** 10.1016/j.xops.2025.100732

**Published:** 2025-02-04

**Authors:** Maria Cristina Savastano, Claudia Fossataro, Alessandro Berni, Alfonso Savastano, Valentina Cestrone, Federico Giannuzzi, Francesco Boselli, Matteo Mario Carlà, Mattia Cusato, Francesco Mottola, Riccardo Pirolo, Elena D'Agostino, Ilaria Biagini, Sofia Marcelli, Alessandro Gravina, Mengxi Shen, Clara Rizzo, Caterina Giovanna Valentini, Maria Bianchi, Luciana Teofili, Yuxuan Cheng, Ruikang K. Wang, Philip J. Rosenfeld, Stanislao Rizzo

**Affiliations:** 1 Ophthalmology Unit, Fondazione Policlinico Universitario Agostino Gemelli, IRCCS, Rome, Italy; 2 Ophthalmology Unit, Catholic University “Sacro Cuore”, Rome, Italy; 3 Department of Ophthalmology, Bascom Palmer Eye Institute, University of Miami Miller School of Medicine, Miami, Florida; 4 Department of Ophthalmology, IRCCS San Raffaele Scientific Institute, Milan, Italy; 5 Department Neurofarba, University of Firenze, Firenze, Italy; 6 Ophthalmology, Department of Surgical, Medical and Molecular Pathology and Critical Care Medicine, University of Pisa, Pisa, Italy; 7 Ophthalmology Unit, University of Verona, Verona, Italy; 8 Division of Hematology, Department of Radiological and Hematological Sciences, Catholic University “Sacro Cuore”, Rome, Italy; 9 Department of Bioengineering, University of Washington, Seattle, Washington; 10 Department of Ophthalmology, University of Washington, Seattle, Washington; 11 Consiglio Nazionale delle Ricerche (CNR), Istituto di Neuroscienze, Pisa, Italy

**Keywords:** CB-PRP, Cord blood platelet-rich plasma, Dry age-related macular degeneration, En face OCT, Geographic atrophy

## Abstract

**Purpose:**

Intravitreal injections (IVIs) of umbilical cord blood platelet-rich plasma (CB-PRP) were investigated to assess their safety and efficacy in slowing the progression of atrophy in eyes with late-stage dry age-related macular degeneration (AMD).

**Design:**

Randomized, controlled, prospective study.

**Subjects:**

Patients with AMD aged >65 years and diagnosed with bilateral geographic atrophy were enrolled.

**Methods:**

One eye of each subject received the treatment of intravitreal CB-PRP 0.05 ml, while the fellow eye received a sham injection. Atrophic areas were identified as large choroidal hypertransmission defects (hyperTDs) on en face subretinal pigment epithelium slabs (64–400 μm beneath Bruch's membrane) obtained 0.321 from swept-source OCT angiography scans. The main outcome was the mean annualized growth rate of the square root transformed area measurements in both treated and nontreated eyes.

**Main Outcome Measures:**

The mean ± standard deviation (SD) square root hyperTD area in the treated eyes was 3.30 ± 0.99 mm at baseline and 3.49 ± 0.98 mm after CB-PRP (IVI). In nontreated eyes, the mean square root hyperTD area was 2.96 ± 0.94 mm at baseline and 3.18 ± 0.94 mm after sham injections.

**Results:**

Twenty-six eyes of 13 patients were included. In treated eyes, the mean ± SD best-corrected visual acuity (BCVA) was 48.92 ± 16.33 letters at baseline and 51.46 ± 12.27 letters at last follow-up. In untreated eyes, BCVA was 67.69 ± 10.89 letters at baseline and 65.38 ± 10.34 letters at last follow-up. The mean follow-up was 258.46 ± 97.54 days. In both groups, no statistically significant difference was observed between the baseline and final BCVA. For treated eyes, the mean annualized growth rate (square root) was 0.275 mm and for nontreated eyes it was 0.321 mm. The annualized growth rate of the hyperTDs in treated eyes was 14.5% lower than that in nontreated eyes (*P* = 0.007). No adverse events were recorded.

**Conclusions:**

These preliminary data suggest that intravitreal CB-PRP injections might be safe and effective in slowing the progression of atrophy in AMD. Extended follow-up and larger sample sizes are needed to confirm our findings and determine the optimal treatment regimen for this novel treatment option in late-stage dry AMD.

**Financial Disclosure(s):**

Proprietary or commercial disclosure may be found in the Footnotes and Disclosures at the end of this article.

Age-related macular degeneration (AMD) is the leading cause of irreversible central vision loss among the elderly in industrialized countries.^1^^,^^2^ It is estimated that by 2050, 61 million people will be legally blind, 474 million will have moderate to severe vision impairment, and 360 million will experience mild vision impairment due to AMD.^3^

The nonexudative form of AMD, usually referred to as dry AMD, is the most prevalent subtype of the disease, with its late stage represented by geographic atrophy (GA).^4^ Historically, color fundus imaging has been the reference imaging modality for diagnosing and defining all stages of AMD. More recently, fundus autofluorescence has been used for detecting and quantifying GA lesions, highlighting atrophic areas as dark regions due to the loss of retinal pigment epithelium.^5^^,^^6^ However, OCT is now considered the gold standard for imaging the onset and progression of macular atrophy.^6^ OCT imaging directly identifies both the retinal pigment epithelium atrophy and photoreceptor degeneration associated with GA.^6^^,^^7^ The identification and measurement of all atrophic areas can be performed by combining conventional OCT B-scans with en face images of volumetric OCT scans.^8^, ^9^, ^10^ These atrophic areas can be identified as large choroidal hypertransmission defects (hyperTDs) on en face subretinal pigment epithelium (subRPE) slabs ranging from 64 to 400 μm beneath Bruch's membrane.^11^, ^12^, ^13^, ^14^

Significant progress has been made in understanding the development and progression of GA, with particular attention to the role of inflammation, especially within the complement cascade.^15^ The identification of genetic risk alleles in the complement factor H locus suggested an important role of complement dysregulation in the development of AMD, and the recent success of slowing the growth of GA with the use of intravitreal complement inhibitors further supports the importance of inflammation in AMD.^16^, ^17^, ^18^, ^19^, ^20^, ^21^ These novel treatments for GA have demonstrated a reduction in the atrophic growth by approximately 20% in the injected eyes. Despite this encouraging result, the novel drugs did not provide clear benefits in terms of visual function.

Another potential strategy to reduce low-grade inflammation in the macula would be to use injections of umbilical cord blood platelet-rich plasma (CB-PRP).^15^ Platelet-rich plasma therapy gained attention in the 1970s to treat patients with thrombocytopenia, and since then, it has been used in many fields, including sports injuries, plastic surgery, dermatology, medical aesthetics, and cardiac surgery.^22^, ^23^, ^24^, ^25^

Additionally, CB-PRP proved to promote the differentiation and proliferation, as well as the healing, of damaged cells.

Cord blood platelet-rich plasma in GA may potentially deliver trophic and regenerative factors that could better support photoreceptors experiencing oxidative stress or degeneration. Cord blood platelet-rich plasma contains several neurotrophic factors, such as nerve growth factor and fibroblastic factor. The neuroprotective and antioxidant effect of CB-PRP may favor photoreceptors' survival in the degenerative process of dry AMD.^26^, ^27^, ^28^

Recently, Rizzo et al^29^ reported on the safety of subretinal injections of CB-PRP in patients with retinitis pigmentosa and AMD-associated GA. In AMD eyes, preliminary data demonstrated a mean gain of 5 ETDRS letters 6 months after treatment with a gain of 2 letters at 12 months.

In view of the above-mentioned results, both in terms of efficacy and safety, our group has proposed a novel therapeutic protocol based on intravitreal injections (IVIs) of CB-PRP in patients with AMD with GA. The safety of intravitreal CB-PRP injections has been previously demonstrated.^30^ In the current study, we further evaluated both the safety and efficacy of this novel therapeutic strategy by randomizing patients with AMD with bilateral GA to receive intravitreal CB-PRP in the study eye and sham injections in the fellow eye, and the growth rates of the atrophic areas were analyzed using en face OCT imaging.

## Methods

This interventional single-center study was approved by the Ethical Committee of Fondazione Policlinico Universitario Agostino Gemelli, Rome, Italy, with registration number ID: 4995. The clinical trials.gov registration was applied with the number NCT05706896. A written informed consent was obtained from each patient. The study was conducted in accordance with the 1976 Declaration of Helsinki and its later amendments.

This masked, randomized, controlled, open-label, prospective experimental study was conducted at Fondazione Policlinico Universitario Agostino Gemelli, Istituto di Ricovero e Cura a Carattere Scientifico, Rome, Italy, between January 2023 and June 2024. Patients diagnosed with AMD were enrolled in the study if they were ≥65 years old and had GA on color fundus imaging in both eyes. Only patients with best-corrected visual acuity (BCVA) between 35 and 65 ETDRS letters in both eyes were included. Exclusion criteria included the presence of any systemic or other ocular diseases in both eyes, such as glaucoma, amblyopia, diabetic retinopathy, uveitis, history of exudative or nonexudative macular neovascularization, and the presence of any significant optic media opacity that could influence the quality of images.

At baseline and subsequent follow-up visits scheduled 3, 6, and 12 months later (±7 days), all enrolled patients underwent a complete ophthalmic evaluation including BCVA assessment, slit-lamp examination, intraocular pressure measurement, fundus examination, color fundus imaging (Eidon, Centervue), and swept-source OCT angiography (Plex Elite 9000, Carl Zeiss Meditec Inc). The swept-source OCT angiography scanning protocol included both 6 × 6 mm and 9 × 9 mm scan patterns centered on the fovea.

Both eyes of each patient were included in the study with 1 eye receiving IVIs of CB-PRP 0.05 ml and the other eye receiving sham injections. If the visual acuity was the same in both eyes, then we decided to randomize the treated eye; otherwise, the eye with the lower BCVA was treated. Enrolled patients were randomized into 3 treatment arms: monthly, every other month (a 2-month interval between injections), or IVIs every 3 months. The 3 substudies were conducted in parallel for 1 year. The safety of the IVI of CB-PRP was examined the day after the procedure with slit-lamp and dilated fundus examinations. If any ocular adverse event (AE) (septic bacterial or fungal endophthalmitis, retinal detachment, proliferative vitreoretinal reaction with retinal traction, secondary glaucoma, ocular phthisis, or rubeosis iridis) was observed, the principal investigator reported it to the medical doctor of Blood Transfusion Service, who recorded the event in the system. No further injections would be administered after an ocular AE.

### Preparation of CB-PRP

Cord blood platelet-rich plasma is a blood component used for nontransfusion purposes, produced according to procedures defined by the Italian legislation on blood components.^31^^,^^32^ The basic materials to produce PRP are units of cord blood collected at the Cord Blood Bank of “Fondazione Policlinico Gemelli, Istituto di Ricovero e Cura a Carattere Scientifico, Rome, Italy.” For the current study, only units unsuitable for transplantation due to insufficient quantity of hematopoietic progenitors and negative for all microbiological screening tests (serology and genome of human immunodeficiency virus, hepatitis B virus, hepatitis C virus, and serological test for syphilis, blood culture for fungi, aerobic and anaerobic bacteria) were used.^33^^,^^34^

**Figure 1:**
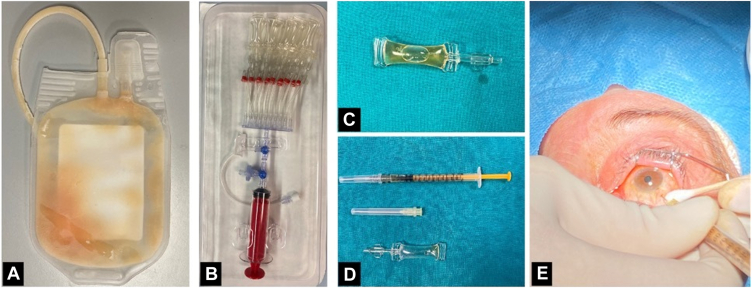
Cord blood platelet-rich plasma syringe preparation: Extraction of CB-PRP from the pool bag to intravitreal injection. **A,** Collection bag of the 15 pools of cord extracts. **B,** Sterile syringe with closed connection system with 20 microvials of 0.2-ml content each. **C,** Microvials filled with 0.2 ml of CB-PRP. **D,** Vial emptied by aspiration of its contents through a millimeter insulin syringe (orange syringe) and ready for intravitreal injection with 30-gauge needle (yellow needle). **E,** Intravitreal injection procedure with needle insertion at 3.5 mm from the limbus in pseudophakic eyes and at 4 mm from the limbus in phakic eyes in the inferotemporal sector 0.05 ml of CB-PRP is injected. CB-PRP = cord blood platelet-rich plasma.

Fifteen blood units constituted the CB-PRP pool. Each unit underwent “soft-spin” centrifugation to obtain PRP in which the platelet concentration was determined and normalized to 1 × 10^9^/L through hard-spin centrifugation followed by the removal of excess platelet-poor plasma. These units of CB-PRP were stored at −80°C before microbiological tests. The risk of having different concentrations of the growth factors in each unit was overcome by thawing and combining the contents of the 15 units into a single sterile bag, specifically designed for the preparation of blood components for nontransfusion use (Fig 1A).

The pooled CB-PRP was then fractionated into 0.2-ml aliquots in sealed sterile vials and then stored again at −80°C until use (Fig 1B, C). All previously-described steps to prepare the CB-PRP pool, as well as the splitting of the aliquots, were carried out using sterile cryogenic blood component bags with sterile connections for transfers of CB-PRP (Fig 1). The aliquot was thawed at room temperature for 30 minutes before administering the IVI (Fig 1D, E).

### IVI of CB-PRP Procedure

The treated eye received local anesthesia with 3 drops of chlorhydrate oxybuprocaine 2 minutes before the IVI. Accurate disinfection of the periocular skin and conjunctival fornix with povidone-iodine 5% followed. Injections were administered 3.5 mm from the limbus in pseudophakic eyes (intraocular lens previously implanted for cataract surgery) or 4 mm from the limbus in phakic eyes (with natural lens), and a precalibrated compass was used to mark the site of injection in the inferotemporal sector.

The IVI of 0.05-ml CB-PRP was performed with a 30-gauge needle. At the end of the procedure, the eye was again disinfected and medicated with steroid and antibiotic eye drops (Fig 1E). The fellow eye was prepared as the injected one. However, it received only a sham injection using the syringe cap located on the conjunctiva for 2 seconds without needle penetration.

### Images Analysis

The efficacy of CB-PRP injections was evaluated by measuring the growth of macular atrophy.

**Figure 2:**
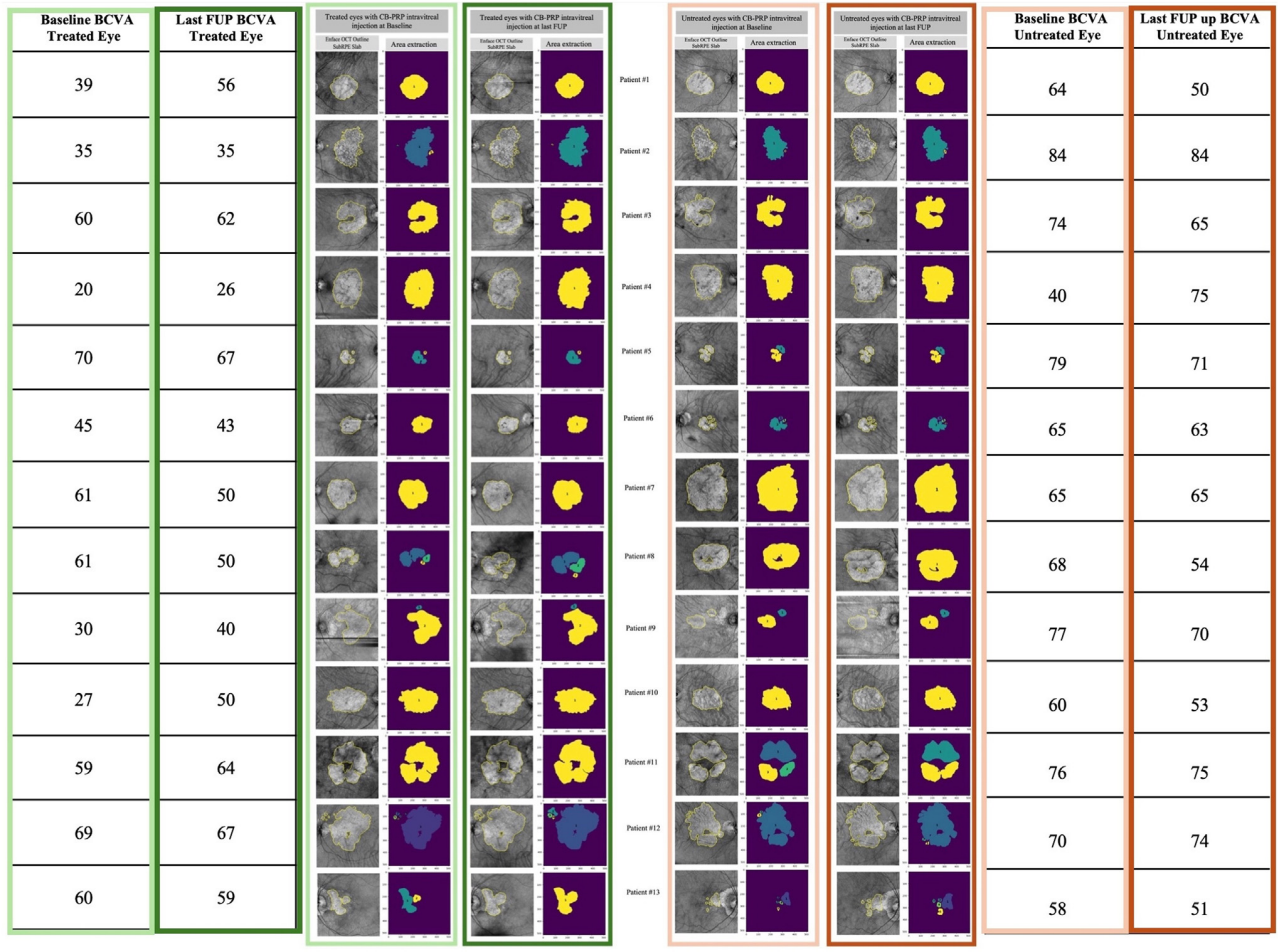
Detection and area measurements of large choroidal hyperTDs on en face SS-OCTA structural images. The left columns show en face subRPE slabs with the outline of hyperTD areas on both treated (green frames) and sham-treated (red frames) eyes. The areas of these outlined regions were subsequently quantified for both eyes at baseline and at the last CB-PRP intravitreal therapy. The right columns display color-coded outlines for each individual hyperTD, allowing for clear differentiation among them. For both treated and untreated groups, BCVA at baseline and the last follow-up was reported. BCVA = best-corrected visual acuity; CB-PRP = cord blood platelet-rich plasma; FUP = follow-up; hyperTDs = hypertransmission defects; SS-OCTA = swept-source OCT angiography; subRPE = subretinal pigment epithelium.

Swept-source OCT angiography scans were performed at baseline and at 1 year after initiation of CB-PRP therapy and graded at the Bascom Palmer Eye Institute (Miami, Florida). Large choroidal hyperTDs were identified as areas of focal brightness with a greatest linear dimension ≥250 μm on en face subRPE slabs positioned from 64 to 400 μm beneath Bruch's membrane, as previously described.^35^ The subRPE slabs were generated primarily using 9 × 9 mm scans. In cases where the quality of the 9 × 9 mm scans was suboptimal, the 6 × 6 mm scans were used instead. The quantitative analysis accounted for differences in scan dimension and the same scan size was used at different time points for comparison for each patient. Two graders (A.B. and M.S.) independently examined all baseline and follow-up visits to identify all large choroidal hyperTDs. Once large hyperTDs were identified on the en face subRPE slabs, B-scans through the lesions were reviewed to confirm the presence of hyperTDs, and each hyperTD was labeled with a unique number. The 2 graders reached a consensus on the identification of each lesion, and any remaining disagreements were adjudicated by a senior grader (P.J.R.). A proprietary, validated, semiautomated algorithm was then used to outline each large hyperTD on the subRPE slabs to obtain the area measurements (mm^2^) for each individual lesion and the total area (Fig 2).^36^^,^^37^ The 2 graders collaboratively and manually checked all the algorithm's outlines and corrected them using a built-in editing tool within the algorithm, as necessary.

### Statistical Analysis

The clinical and demographic features of the sample were presented by descriptive statistical techniques.

The total area measurements obtained from the algorithm underwent a square root transformation, since this strategy eliminates the influence of baseline lesion size on the test-retest variability of atrophy measurements, as previously described.^38^^,^^39^ For each eye, the annualized area growth was calculated by subtracting the baseline square root area from the follow-up square root area and then annualizing this difference over 365 days. The mean annualized growth rate was then determined for all eyes in each group (treated vs. sham).

Quantitative variable distribution was evaluated by Shapiro–Wilk test. Therefore, quantitative variables will be summed up by mean and standard deviation (SD). Absolute frequencies and relative percentages were used to summarize qualitative data.

Adverse event and safety end points were evaluated only with a descriptive analysis, by absolute frequencies and related percentages.

Statistical comparison between baseline and the last follow-up data was performed using Student paired *t* test with a 95% confidence interval. A *P* value <0.05 was considered statistically significant. *P* value between 0.05 and 0.10 was considered suggestive. A Pearson correlation test was performed to evaluate the existence of any relation between the annualized growth rate of GA and the number of injections. Statistical analysis was performed with the R software version 4.2.0 (CRAN, R Core, 2022).

## Results

Eighteen patients were initially enrolled in the study, but only 16 completed the follow-up. Two patients withdrew from the clinical study due to personal scheduling difficulties. Furthermore, 3 additional patients were excluded because the hyperTD area was larger than the 9 × 9 mm scan, making it impossible to fully quantify it. Finally, 26 eyes of 13 patients were analyzed. Four patients were included in the monthly group, 5 in the every other month subgroup, and 4 in the every 3 months arm. The mean ± SD age of the sample was 74.76 ± 6.71 years, with 62% men (male-to-female ratio of 8:5).

**Figure 3:**
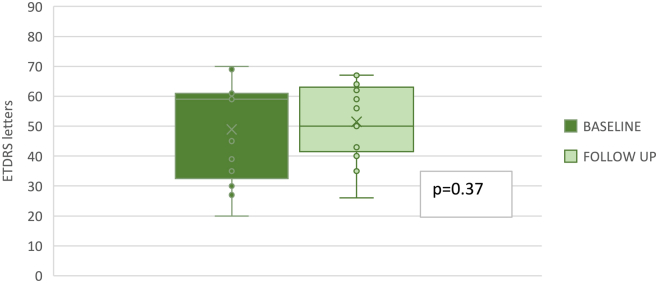
Best-corrected visual acuity in treated eyes. The graph showed the BCVA at baseline and follow-up in injected eyes. No statistically significant difference was observed (*P* = 0.37). BCVA = best-corrected visual acuity.

**Figure 4:**
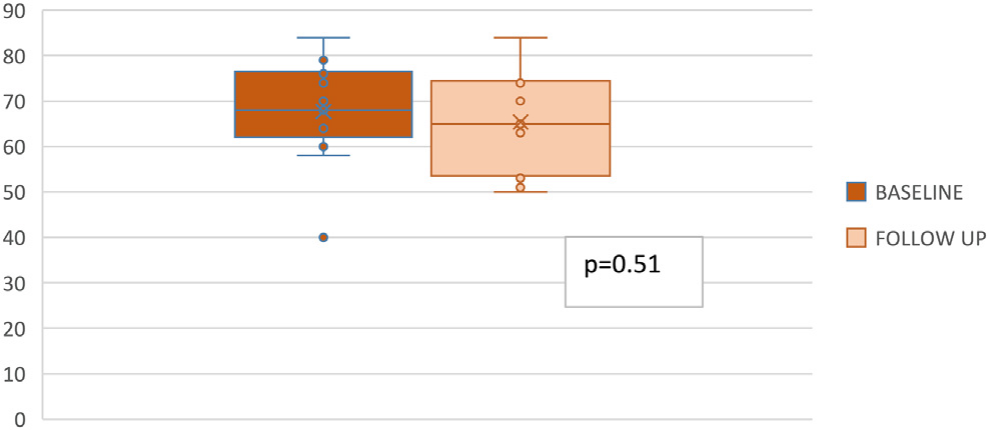
Best-corrected visual acuity in sham eyes. The graph showed the BCVA at baseline and follow-up in untreated eyes. No statistically significant difference was observed (*P* = 0.51). BCVA = best-corrected visual acuity.

In treated eyes, the mean ± SD BCVA was 48.92 ± 16.33 letters at baseline and 51.46 ± 12.27 letters at the last follow-up visit. No statistically significant difference was observed between the baseline and follow-up BCVA values (*P* = 0.37) (Fig 3). The mean follow-up was 258.46 ± 97.54 days. In untreated eyes, BCVA was 67.69 ± 10.89 letters and 65.38 ± 10.34 letters at baseline and at the last follow-up, respectively, with no statistically significant difference (*P* = 0.51) (Fig 4). No significant AEs were observed in treated eyes at any follow-up assessments or during the procedure. Only mild events were reported in our sample, including conjunctival hyperemia in 4 patients that lasted for 1 day, subconjunctival hemorrhage in 3 patients after the IVI, and transitory floaters that lasted for 2 days in 6 patients.

Demographic and clinical data were reported in Table 1.

**Table 1:** Demographic and Clinical Data: Demographic and Clinical Data, Including BCVA at Baseline and Last Follow-Up, and Mild Adverse Events Reported in Both Treated and Untreated Groups

Characteristics	Values
Enrolled Patients	13
Age (mean ± SD)	74.76 ± 6.71
Male:female ratio	8:5
Baseline BCVA (letters) (mean ± SD)	
Treated	48.92 ± 16.33
Untreated	67.69 ± 10.89
Last follow-up BCVA (letters) (mean ± SD)	
Treated	51.46 ± 12.27
Untreated	65.38 ± 10.34
Mild adverse events	
Conjunctival hyperemia	4
Subconjunctival hemorrhage	3
Transitory floaters	6

BCVA = best-corrected visual acuity; SD = standard deviation.

The mean ± SD hyperTD area in treated eyes was 11.84 ± 6.35 mm^2^ and 13.10 ± 6.88 mm^2^ at baseline and the last follow-up visit, respectively. After square root transformation, the mean hyperTD area in treated eyes before injections was 3.30 ± 0.99 mm and 3.49 ± 0.98 mm after injections (Table 2). In treated eyes, the mean annualized growth rate of hyperTD area (square root) was 0.275 mm.

**Table 2:** Annualized Growth Rates: Annualized Growth Rates of Square Root Transformed Hypertransmission Defect Area and Difference in GA Growth Rate, and BCVA at Baseline and Last Follow-Up for All Included Eyes

Patient	Treated Eye	IVI Frequency	Baseline BCVA Treated Eye	Last Follow-Up BCVA Treated Eye	Baseline BCVA Untreated Eye	Last Follow-Up BCVA Untreated Eye	Annualized Growth (mm) En Face OCT Treated Eye	Annualized Growth (mm) En Face OCT Untreated Eye	GA Growth Rate Difference between Treated and Untreated Eyes
#1	LE	Monthly	39	56	64	50	0.116994811	0.198955689	0.081960878
#2	LE	ETM	35	35	84	84	0.152089849	0.169883357	0.017793509
#3	RE	ETM	60	62	74	65	0.307617599	0.333511502	0.025893904
#4	LE	Monthly	20	26	40	75	0.664480714	0.686966192	0.022485479
#5	RE	EOM	70	67	79	71	0.101576572	0.105660371	0.004083799
#6	RE	ETM	45	43	65	63	0.081735141	0.193255924	0.111520782
#7	LE	Monthly	61	50	65	65	0.313582835	0.39995176	0.086368925
#8	LE	EOM	61	50	68	54	0.450471276	0.43870877	−0.011762506
#9	LE	EOM	30	40	77	70	0.121063278	0.108895332	−0.012167946
#10	RE	EOM	27	50	60	53	0.181643094	0.221466694	0.039823599
#11	RE	Monthly	59	64	76	75	0.259217715	0.318830261	0.059612547
#12	LE	ETM	69	67	70	74	0.437279318	0.451658152	0.014378834
#13	LE	EOM	60	59	58	51	0.382121849	0.548139804	0.166017955

BCVA = best-corrected visual acuity; EOM = every other month; ETM = every three months; GA = geographic atrophy; IVI = intravitreal injection; LE = left eye; RE = right eye.

In untreated eyes, the mean ± SD area of atrophy was 9.64 ± 5.65 mm^2^ and 10.97 ± 6.18 mm^2^ before and after the sham injection, respectively. After square root transformation, the mean atrophic area was 2.96 ± 0.94 mm at the baseline and 3.18 ± 0.94 mm after the sham injections (Table 2). In nontreated eyes, the mean annualized growth rate of hyperTD area (square root) was 0.321 mm.

The annualized growth rate (square root) of GA in treated eyes was approximately 14.5% smaller than that in nontreated eyes. The paired *t* test for comparing the annualized growth rates (square root) between the 2 groups resulted in a *P* value of 0.007.

The Pearson test to evaluate the correlation between the number of injections and the annualized growth rate showed no significant correlation (*P* = 0.44; *r* = −0.20).

## Discussion

In recent years, novel treatment strategies have been developed with the purpose of reducing the growth rate of GA in dry AMD. However, no efficacy was observed with eculizumab, lampalizumab, sirolimus, and tesidolumab.^40^, ^41^, ^42^, ^43^ In 2023, the US Food and Drug Administration approved 2 drugs delivered by IVIs, pegcetacoplan and avacincaptad pegol, as treatments for dry AMD.^44^^,^^45^ In the phase II study with pegcetacoplan, the growth of GA was decreased by 29% with monthly injections and by 20% with every-other-month injections,^17^ while in the phase III studies, the GA annualized growth rate decreased by <20%. ^44^^,^^45^

In OAKS, a significant slowing down of GA growth was observed with pegcetacoplan monthly (21%) and pegcetacoplan every other month (16%) after 1 year. In DERBY, only a trend was observed at 12 months, but without reaching the statistical significance. At 2 years, the atrophic growth was slowed by 22% with pegcetacoplan monthly and 18% with pegcetacoplan every other month in OAKS and by 19% and 16% in DERBY.^46^ In GATHER1 and GATHER2 Trials, avacincaptad pegol 2 mg was demonstrated to delay the risk of progression to persistent vision loss compared with sham at 12 months, but this effect was lost at 24 months.^16^

In this study, eyes undergoing CB-PRP IVIs demonstrated a significant reduction in the growth rate of atrophy over time compared with fellow eyes treated with sham injections. Using en face OCT imaging, our findings show that intravitreal CB-PRP slows down the progression of GA. En face OCTA subRPE slabs showed that the mean annualized growth rate of the hyperTD area decreased by 14.5% in the study eyes compared with nontreated fellow eyes.

Regarding BCVA, we observed a relative mean gain of 2.5 letters in treated eyes at the last follow-up and a mean loss of 2.3 letters in the sham subgroup. However, it did not result in statistically significant results in both groups. Thus, CB-PRP appears to slow down the progression of atrophy without any concomitant visual improvement, similar to the current US Food and Drug Administration-approved complement inhibitors. Although this finding was a limitation of our study, it should be considered that the small sample size and the fact that the eye with lower visual acuity was treated may have influenced our results.

Intravitreal injections of CB-PRP also appear to be a safe procedure with no local or systemic side effects as observed in both our previous study and the current study.^30^ Of note, the risk of IVIs procedure should always be considered. Given the possibility of late AEs, follow-up will continue for 1 year after the end of the study. However, the absence of any side effect at the last follow-up for each patient was encouraging. A larger sample size would help to confirm these relevant data.

The rationale for the use of CB-PRP in dry AMD is based on its regenerative effect, which was already described by Valentini et al.^47^ Indeed, CB-PRP is a reservoir of several growth factors that may be of support to the outer retina and choriocapillaris, which are typically impaired in this condition.^48^

The proteomic analysis of the cord blood pool revealed the presence of >200 types of proteins involved in the innate and specific immune response, which plays a major role in dry AMD, as already mentioned.^49^^,^^50^ Furthermore, CB-PRP was shown to be highly rich in proteins fundamental for retinoid metabolism and the phototransduction process, potentially providing support for photoreceptors. Among the other detected factors, it is important to mention the presence of high doses of antioxidants, which may defend the DNA and cell membranes from reactive agents.

The choice to use IVIs rather than subretinal injection, as in our previous study, was based on the lower risk of IVI procedure. In addition, as dry AMD is a chronic and degenerative disease, there is a constant need for trophic substances. The regenerative substances in the CB-PRP require repetitive administrations to be active.^51^ Therefore, this novel approach allowed for the assessment of the efficacy of repeated administrations of CB-PRP in GA eyes.

Our study had several limitations, the most significant being its small sample size and the short follow-up time, that do not allow to make firm conclusions about CB-PRP efficacy. However, this was a pilot study examining a novel procedure that had never been described previously and that resulted safe at 1-year follow-up. To circumvent this limitation, future plans include a multicenter clinical trial with a longer treatment duration and follow-up. Recently, the Ministry of Health has awarded a grant, according to the National Recovery and Resilience Plan (PNRR-MCNT2-2023-12377045), for this project that will allow us to extend the treatment to more patients, including additional study centers. Another limitation of this study was our inability to perform a subgroup analysis according to monthly, every other month, or every 3 months arm due to the small sample size. However, this type of analysis will be possible when all enrolled patients complete their follow-up. Given the novelty of the procedure, we chose to perform the CB-PRP injection in the eye with lower visual acuity to minimize the risk of functional impairment, in the case that the treatment resulted to be unsafe. This selection may have biased our outcomes since the baseline lower visual acuity was likely related to greater GA area or foveal involvement. To reduce the influence of baseline GA area, we applied the square root transformation, which partially negates this issue. However, as reported by Shen et al,^52^ it should be considered that a correlation between baseline GA area and square root area persists. Another limitation was that we did not consider the topography of the lesions relative to the fovea at baseline, which may influence their progression.^53^

Other possible difficulties that we may encounter in the future may be the shortage of CB-PRP due to reduced cord donations. To approach this issue, our research group is organizing information campaigns to raise awareness, and we hope to obtain more umbilical cord donations in the future. In view of the necessity of multiple injections, a potential concern may be the repercussions on the care plan. However, if we demonstrate that every other month and every 3 months administration are able to generate the same effect in slowing disease progression, then this may reduce the effort for both the patient and the health care providers.

Intravitreal CB-PRP injections could provide treatment for those patients who do not yet have access to pending US Food and Drug Administration-approved treatments. Should preliminary data be confirmed, intravitreal cord injections could become a useful alternative strategy for patients with this blinding condition.

We aim to expand this protocol by organizing a multicenter clinical study with a greater sample size and longer follow-up to determine if CB-PRP can further slow the progression of the GA in AMD.
